# Non-typhoidal *Salmonella* soft-tissue infection after gender affirming subcutaneous mastectomy case report

**DOI:** 10.1080/23320885.2023.2185621

**Published:** 2023-03-06

**Authors:** Branden T. Barger, Mikhail Pakvasa, Melinda Lem, Aishu Ramamurthi, Shadi Lalezari, Cathy Tang

**Affiliations:** a School of Medicine, University of California, Riverside, CA, USA; b Department of Plastic and Reconstructive Surgery, University of California, Orange, CA, USA; c School of Medicine, University of California, Irvine, CA, USA; d Medical College of Wisconsin, Affiliated Hospitals, Inc., Graduate Medical Education, Milwaukee, WI, USA

**Keywords:** Chest masculinization, top surgery, transgender health, salmonella, case report

## Abstract

We present a case of a 32-year-old transgender male who underwent chest masculinization, complicated by purulent soft tissue infection of bilateral chest incisions. Cultures tested positive for non-typhoidal *Salmonella*, methicillin-resistant *Staphylococcus aureus*, and *Pseudomonas aeruginosa*. Herein, we discuss multiple factors contributing to the complexity of treating this patient’s clinical course.

## Introduction

Gender affirming surgery (GAS) is a growing branch of urologic, gynecologic, and plastic surgery whose procedures are essential and confirming to many patients experiencing gender dysphoria [1]. These patients include transgender and other patients who identify with gender(s) that are not congruent with their gender assigned at birth (*e.g.* gender non-conforming, genderqueer, or nonbinary). GAS procedures are highly individualized and are broadly categorized into facial, chest, and genital surgery, with the aim of achieving a physical appearance that is more congruent with someone’s affirmed gender [1]. This may include, but is not limited to, facial feminization or masculinization, chest augmentation or masculinization (mastectomy), hysterectomy or oophorectomy, and vaginoplasty or phalloplasty.

It has been shown that GAS is associated with improved psychosocial outcomes and mental health in this patient population [2,3]. However, as with every operation, GAS comes with the risk of complications such as surgical site infection (SSI), which can cause patients and physicians significant distress from pain, possible progression to sepsis, reoperations, and long antibiotic courses. While modern precautions and aseptic technique contribute to lower rates of SSI, 2 to 4% of all patients undergoing surgery still develop SSI and the etiology of these infections is often multifactorial [4]. In addition, transgender patients often present with complex social and medical histories potentially further complicating the treatment course of GAS-related SSIs. Successful holistic care and recovery of transgender patients requires a unique multidisciplinary approach, with support from their community and peers as well as input from endocrinologists, surgeons, infectious disease specialists, and mental health professionals.

Herein, we present a case of gender affirming chest masculinization (referred to colloquially as top surgery) consisting of a bilateral mastectomy, which was complicated by post-operative purulent soft tissue infection of bilateral chest incisions with cultures growing non-typhoidal *Salmonella*, methicillin-resistant *Staphylococcus aureus* (MRSA), and *Pseudomonas aeruginosa*. We discuss multiple factors, including medications, comorbidities, and social factors that may have contributed to this patient’s course and demonstrate some of the complexity in treating transgender patients.

## Patient history

A 32-year-old transgender male presented to clinic interested in gender affirming chest masculinization. After confirming he met all WPATH criteria^1^ for surgical treatment [5], he was scheduled for a bilateral double opposing incision subcutaneous mastectomy with free nipple grafting given the large and ptotic nature of his breasts.

### Medical history

Prior to surgery, the patient had been taking 100 mg Depo-Testosterone weekly for five years as hormone replacement therapy, which was continued through his surgical treatment course. The patient had a history of SARS-COVID-2 eight months prior to his operation, which did not require hospitalization. The patient additionally reported a significant history of genitourinary infections, including recurrent urinary tract infections, pyelonephritis, pelvic inflammatory disease, syphilis, and genital herpes. Notably, he was hospitalized for pelvic inflammatory disease and neurosyphilis six months prior to the operation. The patient had no history of immunosuppressant medications or immunosuppression, confirmed with a negative pre-operative immunology workup, including HIV/AIDS testing. He also had a diagnosis of obesity, with a body mass index (BMI) of 32. Patient endorsed allergies to penicillin and fluoroquinolone antibiotics which included development of an urticarial rash and throat swelling, respectively.

### Surgical history

Surgical history included prior repair of his right anterior collateral ligament, appendectomy, cholecystectomy, and ganglion cyst removal. The patient denied adverse events from any of his previous procedures.

### Psychiatric history

Psychiatric history was significant for generalized anxiety disorder and bipolar disorder type I managed with bupropion, hydroxyzine, oxcarbazepine, and quetiapine. However, a few weeks prior to the surgery, the patient was transitioned from quetiapine to risperidone due to tachycardia thought to be associated with the quetiapine.

### Family history

Family medical history was non-contributory to this case.

### Social history

Social history at the time of pre-operative appointment was significant for nicotine vaping and prior cigarette smoking. The patient was counseled on nicotine cessation during his initial surgical consultation.

## Clinical course

**Figure 1.**
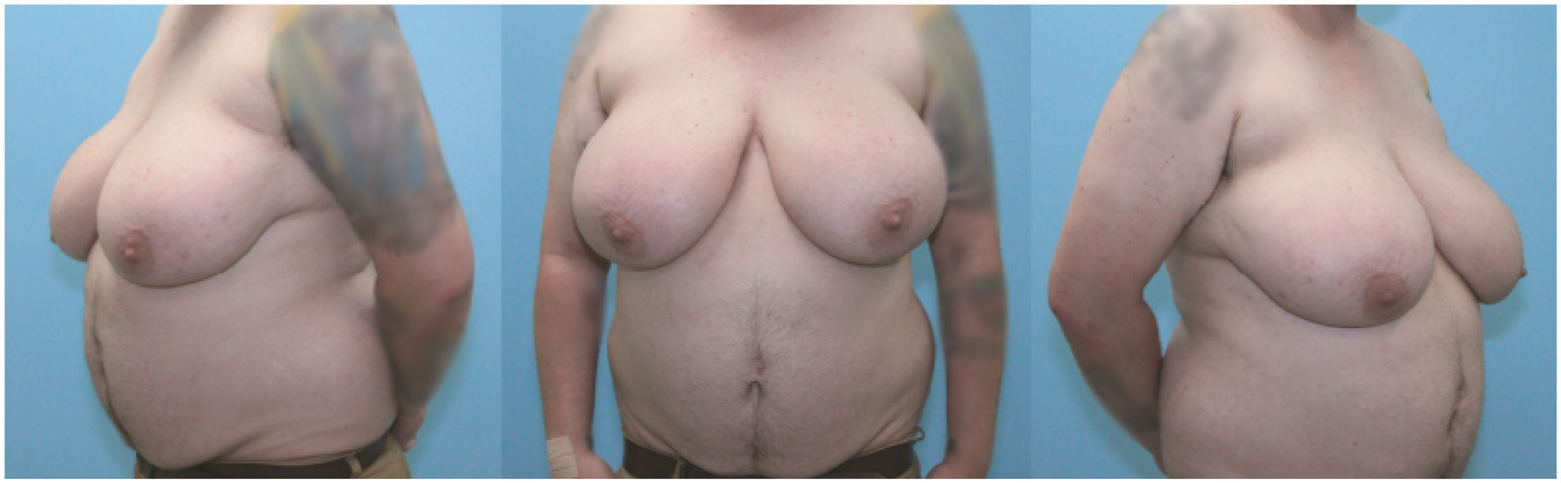
Pre-operative photos of 32-year-old transgender male.

At his pre-operative appointment, the patient confirmed he was still taking masculinizing hormone replacement therapy and he denied any adverse effects from his existing psychiatric medication regimen. The patient also confirmed cessation of all nicotine products over the previous two weeks with little difficulty. Pre-operative physical exam (Figure 1) revealed an obese body habitus, diffuse acne, and multiple, scattered seborrheic keratoses.

**Figure 2.**
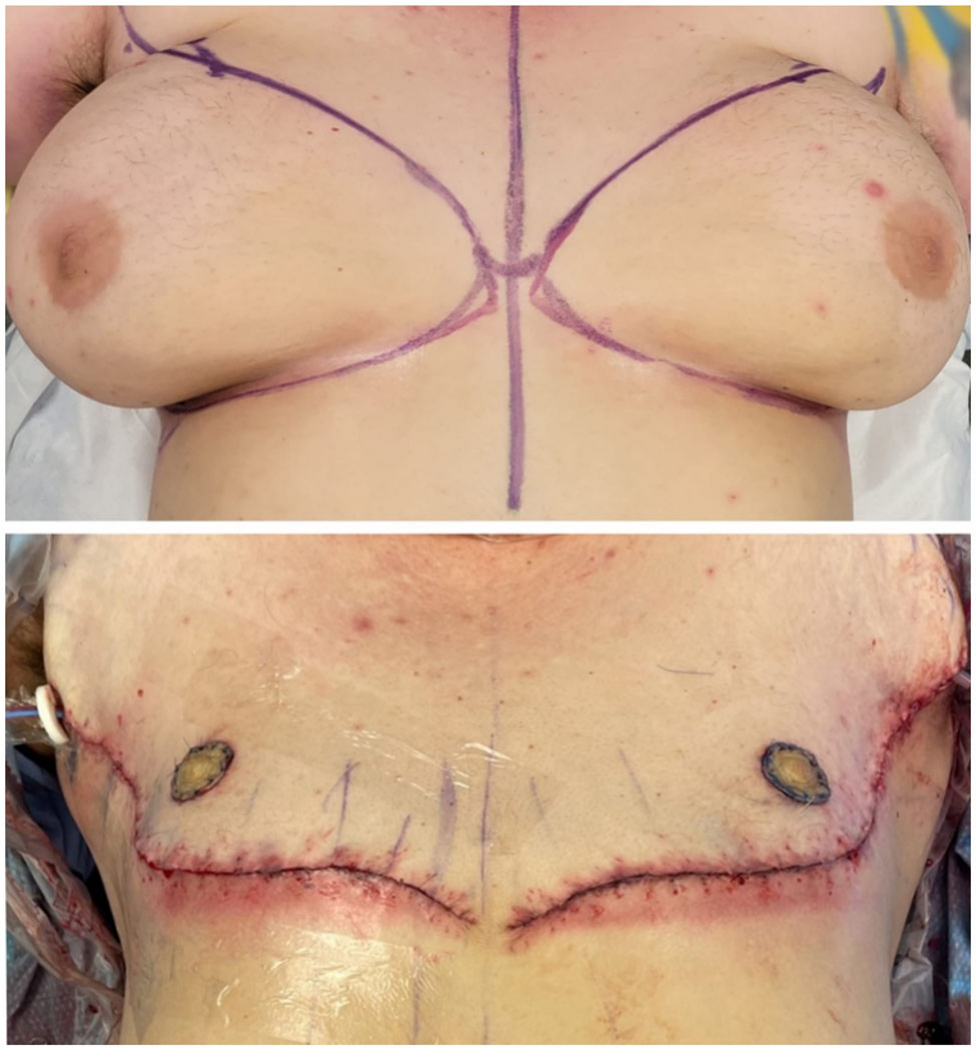
Pre-operative patient surgical markings and immediate post-operative result after double opposing mastectomy with free nipple graft, POD 0.

Prior to the start of surgery, the patient was given a pre-operative dose of 900 mg Clindamycin due to his penicillin allergy and his surgical site was cleaned with chlorohexidine. A bilateral double opposing incision subcutaneous mastectomy with free nipple grafting was performed retaining some amount of breast tissue to avoid excessive thinning of the chest flaps and to match his existing body habitus. Figure 2 outlines pre-operative surgical markings and immediate post-operative results following incision closure. Negative pressure incisional vacuum seal dressings were applied at the time of the operation to the incisions and as a bolster for the nipple grafts. With no immediate post-operative complications, the patient was discharged home on post-operative day 0 with pain medication and a seven-day course of oral Clindamycin.

**Figure 3.**
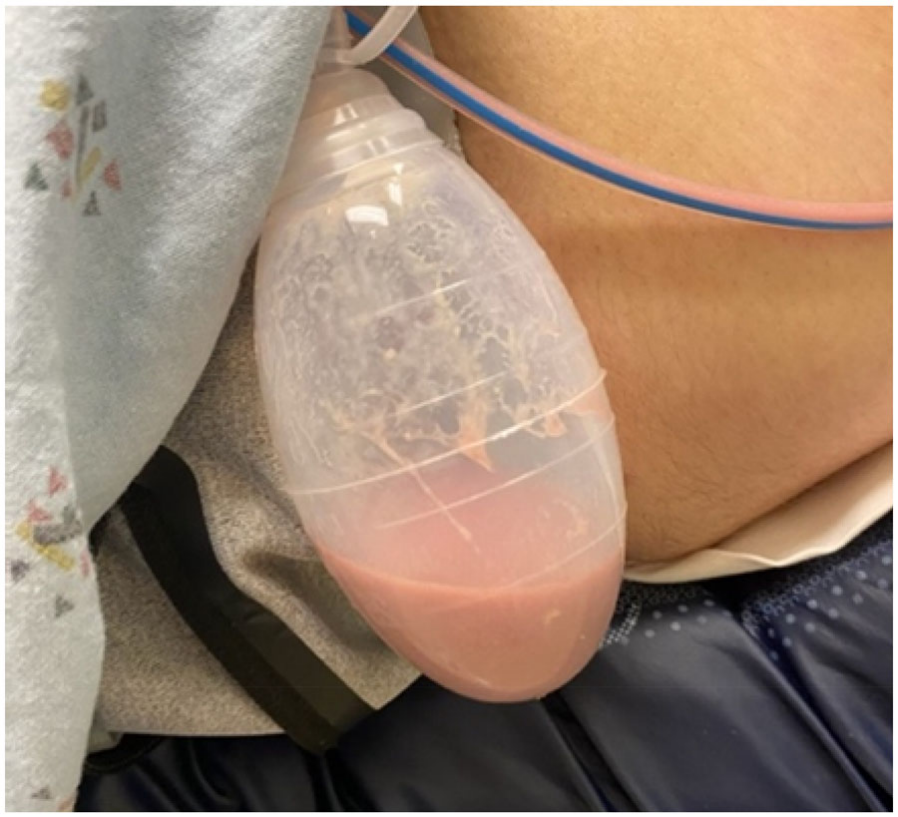
Purulent drainage from subcutaneous Jackson-Pratt drain, POD 7.

On post-operative day 7, he presented to the clinic for his initial post-operative visit. At this time, he was found to have frank purulence in the bilateral subcutaneous Jackson Pratt drains (Figure 3), as well as exquisite tenderness and induration of the bilateral chest incisions and axillae characteristic of cellulitis. While afebrile, he met sepsis criteria with a heart rate of 150, a white blood cell count of 22,900/µL, and known source of infection. The patient was subsequently admitted, given empiric IV broad-spectrum antibiotics, cefepime and vancomycin, and scheduled for washout and debridement of bilateral chest wounds. Cefepime was chosen over more routine broad-spectrum antibiotics, such as fluoroquinolones, due to patient’s previous documented adverse reaction of throat swelling to ciprofloxacin.

**Figure 4.**
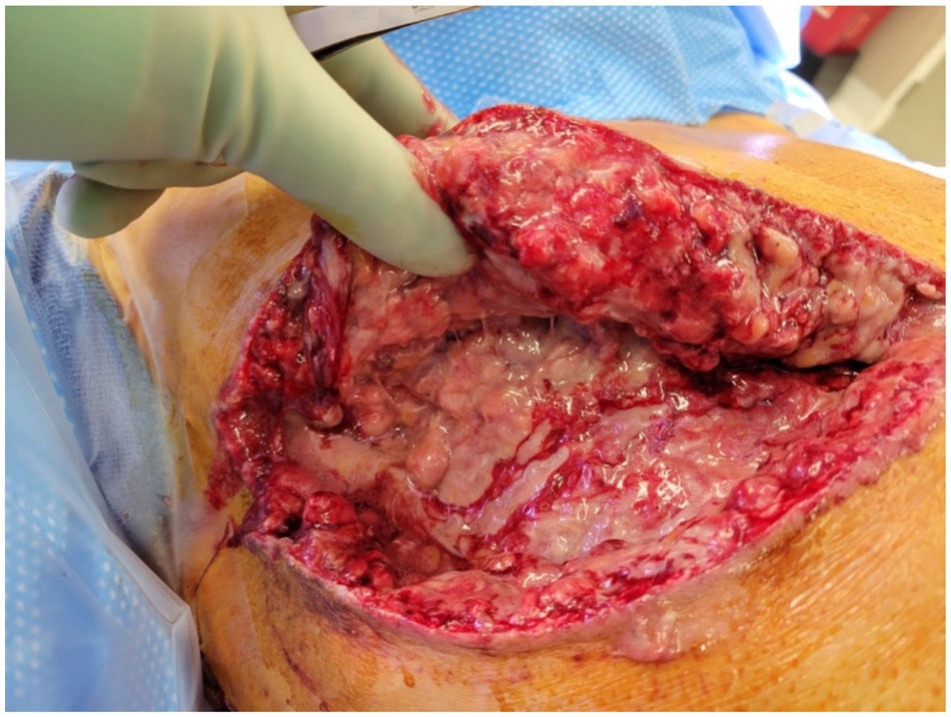
Intra-operative examination of widely disseminated milky fluid within the breast pocket during first washout procedure.

During his initial washout, our patient had an extensive layer of milky, purulent fluid noted throughout the entire surgical dissection plane (Figure 4). This milky fluid was also suspected to be related to galactorrhea. Intraoperative cultures were taken during this initial washout and debridement, and both mastectomy incisions were left partially opened for local wound care.

Cultures grew methicillin-resistant *Staphylococcus aureus* (MRSA) in two out of two cultures*, Pseudomonas aeruginosa* in one out of two cultures, and *Salmonella* Montevideo in one out of two cultures. *Pseudomonas* and *Salmonella* were pan-susceptible, while the MRSA isolate was trimethoprim/sulfamethoxazole and vancomycin susceptible. Following culture results, infectious disease was consulted who suggested we start the patient on two weeks of intravenous antibiotics. A PICC line was placed, and the patient was continued on cefepime and switched from vancomycin to trimethoprim/sulfamethoxazole.

Given intraoperative findings and clinical suspicion of possible galactorrhea, as well as the patient’s recent medication switch to risperidone, additional laboratory testing was performed to measure the patient’s prolactin level. On post-operative day 9, the patient’s prolactin was found to be elevated to 56.5 ng/mL (reference range: 3.3–26.7 ng/mL). After consultation with both endocrinology and psychiatry services, the patient was switched from risperidone to olanzapine.

While admitted, dermatology was also consulted and recommended a standard seborrheic dermatitis treatment plan for his existing skin findings as a method to resolve his discomfort as well as address skin bacterial colonization.

**Figure 5.**
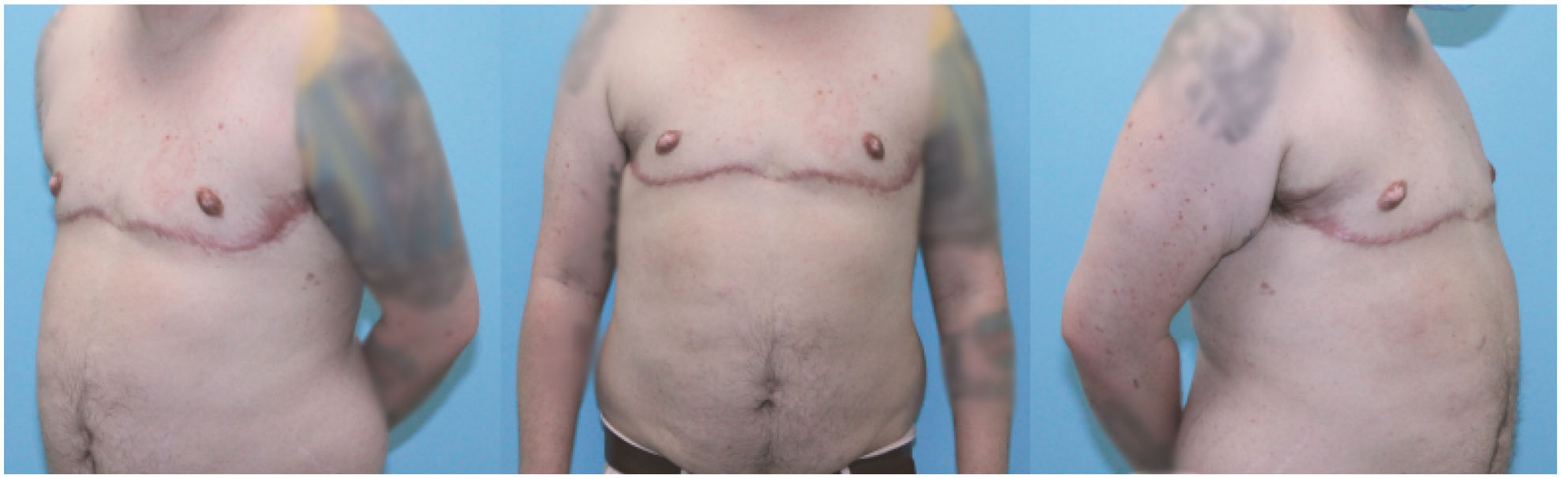
Final post-operative result after 6 months.

After three total irrigation and debridement procedures on post-operative days 7, 13, and 16, the patient’s wounds were sufficiently clean for closure over drains. On post-operative day 19, the patient was discharged with a referral for Home Health Nursing to assist with his continued IV antibiotic regimen of cefepime and trimethoprim/sulfamethoxazole, 2000 mg every 8 hours and 2 tabs 800–160 every 12 hours, respectively. Final 6-month post-operative surgical results are shown in Figure 5.

## Discussion

Gender affirming surgery is an essential procedure that plastic and other surgeons are providing in increasing numbers. This relatively new field has a rapidly enlarging literature base to draw from, but there remain gaps illustrating the vast diversity of this patient population. Gender affirming medical care, including hormone therapy and surgical intervention, has been shown to improve gender dysphoria and quality of life, and decrease rates of depression, anxiety, and other health comorbidities among transgender patients [2,3,6]. Transgender patients are a medically underserved population and experience higher rates of mental and physical illness attributed to excess minority stress, lack of access to medical care, and interpersonal and societal stigmatization [3,7]. Surgeons should be aware of the multiple social, medical, psychiatric, and surgical factors at play when performing surgery on and working with transgender patients. Our case highlights these interactions in a patient with a complex past medical and psychiatric history who developed a significant post-operative infection requiring multiple reoperations.

The overall complication rate of chest masculinization varies widely in the literature from around 11–50%, comparable to other non-gender affirming mastectomy procedures [8–12]. However, chest masculinization is still a relatively safe procedure and re-operation rate ranges from 0–9% [10,13–15]. Studies show that the most frequent complication of the operation is wound healing issues, especially of the nipple areolar complex (NAC), cited from 0 to 18.5% in the literature [9,11–14]. Perez-Alvarez et al. and Pittelkow et al. examined rates of complication after chest masculinization in obese transgender men (defined as BMI >30) and found overall complication rates of 31.5% and 6.9%, respectively [13,16]. Notably, both papers showed relatively more infections in obese patients undergoing these procedures, with rates of 2.9% and 4.8%, respectively. Pittelkow et al. additionally showed that increasing body mass index (BMI) was associated with an increasing rate of post-operative infection [16]. Furthermore, smoking and nicotine use has been shown to be associated with infection and delayed wound healing [17,18]. Studies have shown decreased complications with smoking cessation prior to surgery, with larger effects seen when patients quit farther out from surgery [19]. Relatively recent smoking cessation and high BMI both may have contributed to our patient’s risk profile for post-operative SSI.

*Non-typhoidal Salmonella* is a gram-negative facultative anaerobe known to cause food-borne illness and gastroenteritis. It is an extremely rare cause of soft tissue infection and the literature on the subject is sparse. The strain found in our patient, the Montevideo serovar of *Salmonella enteritica,* is also exceptionally rare and known to cause gastrointestinal disease such as enteritis in humans usually from infected food sources [20,21]. Reports of the Montevideo serotype causing soft tissue infections are not well documented in the literature [22]. However, at least three reports include mention of breast or chest wall infections by *Salmonella*, hinting that the pathogen may have a proclivity for migrating to and colonizing this area of the human body.

In the few reports of soft tissue infections caused by *Salmonella* that exist, most authors point to either hematogenous spread of a gastrointestinal illness or systemic infection in immunocompromised patients (*e.g.* patients positive for HIV/AIDS) as the causal route for this pathogen [23–26]. It is unlikely that either of these routes contributed to our patient’s presentation as he had no prior reports of gastrointestinal illness, nor was he in an immunocompromised state. While our patient had one out of two cultures positive for *Salmonella* Montevideo, it is possible that this was a contaminant. However, the Montevideo serovar is extremely rare in the environment and was likely only one of the bacteria contributing to the infection.

In addition, acne [27] and weight gain [28] are well-documented and common side effects from masculinizing hormone replacement therapy. Though previous literature reports that the most common skin contaminants of acne are *Propionibacterium* and *Cuitbacterium* species, other native skin flora and bacterial species such as *Staphylococcus aureus*, *Streptococcus pyrogenes*, and *Corynecterium* species, have been documented [29]. *Staphylococcus aureus* is also known to be the most common bacterial species in patients with seborrheic dermatitis [30]. Our patient did not undergo pre-operative skin flora culturing, however, it is possible that the microbiologic findings from his SSI wounds, including *MRSA*, *Pseudomonas*, and *Salmonella*, may have been contributors to his dermatologic concerns prior to surgery as well as later complicated proper surgical wound healing. Prior research has indicated that acne treatment such as benzoyl peroxide prior to surgery can lower rates of SSI [31,32], however specific research is needed to better elucidate how HRT-associated acnes, native skin flora, and bacterial overgrowth may impact successful wound healing following chest masculinization and other gender affirming procedures.

Last, an infrequent, though well-documented complication following breast surgery is galactorrhea and galactocele formation [33,34]. Galactorrhea after breast surgery is thought to be due to increased levels of prolactin from stress as well as from inadvertent stimulation of nerves in the chest wall and breast tissue during surgery [35]. Galactorrhea can also be the result of an undiagnosed pituitary tumor, or a side effect of antipsychotic medications such as risperidone. In the case of antipsychotics, the D_2_-dopamine receptor blocking action of these medications turns off the dopamine-prolactin negative feedback loop leading to hyperprolactinemia [36]. To our knowledge, this phenomena has not yet been described among patients undergoing chest masculinization. The patient had recently transitioned to risperidone, which has the highest reported rates of hyperprolactinemia in the literature [37]. Confirmation of fluid as breast milk is not usually done by laboratory testing, and was not performed in our patient as it was not available at our institution. However, the copious white fluid diffusely present in our patient’s surgical plane along with his elevated prolactin levels raised suspicion for galactorrhea. After discussion with both endocrinology and psychiatry services, risperidone was discontinued and his prolactin levels down trended. It remains unclear, whether our patient’s high prolactin levels contributed to his complicated recovery.

Whether this represents a true case of surgical site infection, or simply contaminated galactorrhea, is yet to be elucidated. Overall, our patient’s SSI following bilateral mastectomy was likely multifactorial. His prior medical and social history, including recent smoking and nicotine use, high BMI, long-term use of masculinizing HRT, existing dermatologic concerns, and recent psychiatric medication changes could have all contributed to and complicated this patient’s recovery following surgery. More research is needed to better understand which, if any, of these factors play an integral role in gender affirming post-surgical outcomes.

## Conclusions

While it has been shown that there is no difference in complications after breast augmentation between cisgender^2^ and transgender patients [38], surgeons should remain cognizant of the marginalized position and increased medical and psychological complexity of our transgender patients and the possible effects that this may have on their surgical outcomes and overall health. Further, additional research is needed to study what the effects of dopamine blocking medications are in transgender patients before, during, and after top surgery, as well as the specific risk factors for complications in this population. Continued research is necessitated to ensure that the field of gender affirming surgery is safe and continually beneficial transgender and gender expansive populations.

## Notes

1 WPATH, the World Professional Association for Transgender Health, is a registered 501(c)(3) non-profit that publishes evidence-based Standards of Care and Ethical Guidelines articulating the psychiatric, medical, and surgical management of patients experiencing gender dysphoria. Criteria mentioned in this article follow WPATH Standards of Care, Version 7, which is available for free download at wpath.org

2 Note: the term *cisgender* is commonly used to describe someone who has a gender identity that aligns with that individual’s sex assigned to them at birth (*i.e.* woman and female; man and male). *Cisgender* is used in the context of this article to describe members of a majority population who do not identify as transgender, gender nonconforming, or gender nonbinary, among others.
